# Effect of *Xiaoyaosan* on major depressive disorder

**DOI:** 10.1186/s13020-015-0050-0

**Published:** 2015-07-19

**Authors:** Lin-Lin Jing, Xiao-Xia Zhu, Zhi-Ping Lv, Xue-Gang Sun

**Affiliations:** Traditional Chinese Medicine Integrated Hospital, Southern Medical University, Guangzhou, Guangdong China; The Key Laboratory of Molecular Biology, State Administration of Traditional Chinese Medicine, School of Traditional Chinese Medicine, Southern Medical University, Guangzhou, Guangdong 510515 China; Nanfang Hospital, Southern Medical University, Guangzhou, Guangdong China

## Abstract

**Background:**

This study aims to evaluate the efficacy of *Xiaoyaosan* (XYS) for treatment of major depressive disorder (MDD) and to review the studies on antidepressant mechanisms of XYS.

**Methods:**

The China Knowledge Resource Integrated Database (1998–2014), VIP Journal Integration Platform (1989–2009), and PubMed (1950–2014) were used to search for and collect scientific publications related to XYS and MDD. Clinical trials for “MDD” and “xiaoyao” were screened. Papers that used the original prescription of XYS for treatment and in combination with Western medicines were included, while papers describing modified XYS were excluded. Four investigators read and screened the resulting publications independently, evaluated the associated scientific results and evidence.

**Results:**

There were no conclusive results to support the efficacy of XYS for treatment of MDD, owing to limited sample sizes, flaws in blinding and randomization, and lack of multi-centered clinical trials. Among the experimental studies on the effects of XYS possible involvement of 5-hydroxytryptamine, hypothalamic–pituitary–adrenal axis function, and neuroinflammation were possibly involved demonstrated.

**Conclusions:**

The effectiveness of XYS for treatment of MDD is uncertain.

## Background

Major depressive disorder (MDD) affects ~16% of the world population [1]. In China, the MDD prevalence is 9% in the general population, and 15–30% of all adolescents are estimated to be affected by the disease [2]. Observations from 245,404 subjects in 60 countries revealed that the 1-year prevalence of ICD-10 depressive episodes alone is 3.2% [3]. The depression comorbidity rate of participants with one or more chronic physical diseases ranges from 9.3 to 23.0% [3]. Common antidepressants include selective serotonin (5-hydroxytryptamine; 5-HT) reuptake inhibitors (SSRIs) and serotonin and norepinephrine reuptake inhibitors. The slow onset of action and limited efficacy of these antidepressants have limited their use and prompted the search for novel strategies or alternative methods to combat the illness [4].

*Xiaoyaosan* (XYS) is a Chinese medicinal formula that comprises Radix Bupleuri, Radix Angelicae Sinensis, Radix Paeoniae Alba, Rhizoma Atractylodis Macrocephalae, Poria, Rhizoma Zingiberis Recens, Herba Menthae, and Radix Glycyrrhizae. XYS alone or combined with antidepressants has been used to treat MDD in China. However, the action mechanisms of XYS on MDD are still unknown.

This study aims to evaluate the efficacy of *Xiaoyaosan* (XYS) for treatment of major depressive disorder (MDD) and to review the studies on antidepressant mechanisms of XYS.

## Search strategies

**Figure 1:**
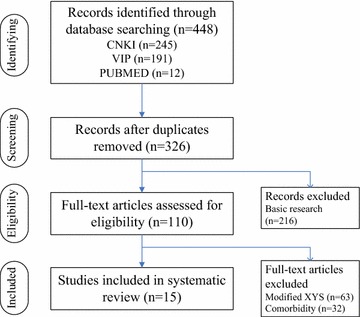
Flow sheet summarizing the study search and selection. Records of basic research were excluded according to the standard of meta-analysis of evidence-based medicine. The full texts on modified XYS were eliminated for the incommensurability of the reports and papers on depression comorbidities were also excluded for our focus on MDD. *CNKI* China Knowledge Resource Integrated Database, *VIP* VIP Journal Integration Platform.

Literature searches were performed with the terms “depression” and “xiaoyao” in the China Knowledge Resource Integrated Database (1998–2014), VIP Journal Integration Platform (1989–2009), and PubMed (1950–2014). After reports describing basic research were excluded, a total of 110 papers on clinical trials were assessed for eligibility. Papers about modified XYS were eliminated from the analysis, because of the difficulty in comparing the efficacies of medicinal formulas based on the incommensurability of the reports [5]. Reports on depression comorbidities, such as postpartum, post-stroke, and post-cancer depression, were also excluded. Papers that used the original prescription of XYS for treatment and/or in combination with Western medicines were included. A total of 15 reports on unmodified XYS and MDD met the inclusion criteria and were evaluated in the present study (Figure 1).

Records of basic research were excluded according to the standard of meta-analysis of evidence-based medicine. The full texts on modified XYS were eliminated for the incommensurability of the reports and papers on depression comorbidities were also excluded for our focus on MDD.

## Efficacy of XYS

### Efficacy of XYS in ameliorating MDD

Regarding the efficacy of XYS in ameliorating MDD, the disease showed significant improvement according to the traditional Chinese medicine curative index [6]. XYS also significantly decreased the Hamilton depression scores in MDD patients [7]. Controlled, randomized, and double-blinded trials showed that the total effective rate of XYS in treating MDD was 91.38% in 58 patients, being significantly higher than the value of 32.69% for the placebo in 52 patients [8]. XYS treatment was associated with a significantly decreased depression inventory score [8] (Table 1).

**Table 1:** Summary of published clinical trials on XYS in the treatment of MDD

Study ID	Sample size	Diagnostic criteria	Trial design				Course (weeks)	Follow-up (months)	Major outcomes				
			Intervention	Control	Randomizer	Blindness			Efficacy by HAMD	Clinical efficacy	Side effects	Relapse	Onset time
Xian et al. [6]	60	CCMD-3	XYS decoction	Fluoxetine	Random number table	Single	6	N/A	N/A	↑	↓	N/A	N/A
Zhang et al. [8]	110	Depression Inventory (DI)	Xiaoyao pill	Placebo	Random number table	Double	8	N/A	N/A	↑ (DI)	N/A	N/A	N/A
Feng et al. [7]	58	CCMD-3	XYS decoction	N/A	N/A	N/A	8	N/A	↑	↑(CGI)	N/A	N/A	N/A

*CCMD* China classification and diagnostic criteria for mental disorder, *HAMD* Hamilton depression scale, *CGI* clinical global impression, *N/A* not available, ↑ significant increase compared with control, ↓ significant decrease compared with control.

### Analysis of XYS-induced efficacy of antidepressants

The total effective rate of XYS–antidepressant combinations in 12 trials was not significantly improved compared with those of other antidepressants in seven trials. However, the cure rate was increased in one of the seven trials. The five remaining trials exhibited improved effective rates in the combination groups. Nine trials demonstrated that XYS reduced side effects, four trials revealed decreased relapse rates, and four trials showed delays in onset time (Table 2).

**Table 2:** Summary of published clinical trials on combinations of XYS and antidepressants in the treatment of MDD

Study ID	Sample size	Diagnostic criteria	Test design				Course (weeks)	Follow-up (months)	Major outcomes				
			Intervention	Control	Randomizer	Blindness			Efficacy by HAMD	Clinical efficacy	Side effects	Relapse	Onset time
Wang et al. [9]	120	CCMD-3	Xiaoyao pill and duloxetine	Duloxetine	N/A	N/A	8	N/A	↑	↑ (CGI)	NS (TESS)	N/A	N/A
Wang et al. [10]	68	CCMD-3	Xiaoyao pill and doxepin	Fluoxetine	N/A	N/A	6	N/A	NS	NS	NS (TESS)	N/A	↑
Nan et al. [11]	61	CCMD-2-R	Xiaoyao pill and imipramine	Imipramine	N/A	N/A	8	6	NS	↑ (cure rate)	↓	↓	N/A
Li et al. [12]	60	CCMD-3	Xiaoyao pill and imipramine	Imipramine	N/A	N/A	8	N/A	NS	NS	↓	N/A	↑
Du et al. [13]	150	CCMD-3	Xiaoyao pill and fluoxetine	Amitriptyline	N/A	N/A	6	N/A	NS	NS	↓ (TESS)	N/A	↑
Ma [14]	30	CCMD-3	Xiaoyao pill and citalopram	Citalopram	Random number table	N/A	8	12	NS	NS	↓ (TESS)	↓	N/A
Chen [15]	61	CCMD-2-R	Xiaoyao pill and amitriptyline	Amitriptyline	N/A	N/A	6	6	↑	↑	↓ (TESS)	↓	N/A
Zhai et al. [16]	24	CCMD-2-R	Xiaoyao pill and doxepin	Doxepin	N/A	N/A	8	N/A	NS	NS	↓ (TESS)	N/A	N/A
Zhang et al. [17]	50	CCMD-3	Xiaoyao pill and fluoxetine	Fluoxetine	N/A	N/A	6	N/A	NS	NS	↓ (TESS)	N/A	↑
Zhang et al. [18]	50	CCMD-2-R	Xiaoyao pill and fluoxetine	fluoxetine	N/A	N/A	3	N/A	N/A	↑ (Zung value)	↓ (TESS)	N/A	N/A
Xia et al. [19]	60	CCMD-3	Xiaoyao pill and citalopram	Citalopram	N/A	N/A	8	N/A	↑	↑	↓ (TESS)	N/A	N/A
Xiang [20]	76	CCMD-3	Xiaoyao pill and paroxetine	Paroxetine	N/A	N/A	12	12	N/A	↑ (SDS)	N/A	↓	N/A

*CCMD* China classification and diagnostic criteria for mental disorder, *HAMD* Hamilton depression scale, *TESS* treatment-emergent symptom side effect, *SDS* self-rating depression scale, *CGI* clinical global impression, *N/A* not available, *NS* no significant difference between intervention and control, ↑ significant increase compared with control, ↓ significant decrease compared with control.

XYS exhibited fewer side effects than antidepressants, but similar effectiveness. The combinations of XYS and antidepressants advanced the onset time and reduced adverse side effects in most of the trials. The methodological quality of most included trials was generally “poor”. So, defects in sample sizes, blindness, and randomization were observed in most of the trials evaluated. Therefore, biases including performance and detection biases could not be ignored [21] (Table 3).

**Table 3:** Quality assessment of included trials

Study ID	Adequate sequence generation	Allocation concealment	Incomplete outcome data	Blinding	Other source of bias	Selective outcome reporting
Xian et al. [6]	Yes	Yes	No	Yes	Unclear	No
Feng et al. [7]	Yes	Yes	Yes	Yes	Unclear	No
Zhang et al. [8]	Unclear	Unclear	No	Unclear	Unclear	No
Wang et al. [9]	Unclear	Unclear	No	Unclear	Unclear	Yes
Wang et al. [10]	Unclear	Unclear	No	Unclear	Unclear	No
Nan et al. [11]	Unclear	Unclear	No	Unclear	Unclear	No
Li et al. [12]	Unclear	Unclear	Yes	Unclear	Unclear	No
Du et al. [13]	Unclear	Unclear	Yes	Unclear	Unclear	Yes
Ma [14]	Yes	Unclear	Yes	Unclear	Unclear	No
Chen [15]	Unclear	Unclear	No	Unclear	Unclear	No
Zhai et al. [16]	Unclear	Unclear	No	Unclear	Unclear	No
Zhang et al. [17]	Unclear	Unclear	No	Unclear	Unclear	No
Zhang et al. [18]	Unclear	Unclear	Yes	Unclear	Unclear	No
Xia et al. [19]	Unclear	Unclear	No	Unclear	Unclear	No
Xiang [20]	Unclear	Unclear	No	Unclear	Unclear	No

## Mechanism of XYS as an integrated model

### Hypotheses for MDD and functions of XYS

5-HT deficiency was the prevailing hypothesis for MDD [22, 23]. SSRIs were widely used, and accounted for about 60–80% of the total market share of antidepressants [24]. XYS upregulated the 5-HT contents in the cerebral cortex of a chronic restraint stress (CRS)-induced rat depression model [25], and increased the 5-HT contents in the hippocampus of rats with postpartum depression [26]. XYS could be a regulator of monoamine neurotransmitters [27].

The hypothalamic–pituitary–adrenal (HPA) axis is governed by secretion of corticotropin-releasing hormone (CRH) from the hypothalamus to activate secretion of adrenocorticotropic hormone (ACTH) from the pituitary gland. Corticoids (cortisol in humans and corticosterone in rodents) are stimulated from the adrenal cortex and interact with their receptors, such as glucocorticoid receptors, for negative feedback control [28]. HPA hyperactivity results from deficits in the negative feedback regulation of the axis based on the failure of glucocorticoid receptor activation to decrease plasma levels of cortisol [29]. XYS downregulated CRH-1 and upregulated CRH-2 expression in the hypothalamus of a CRS-induced depressive rat model [30]. XYS decreased the expression of CRH-1 mRNA in paraventricular nuclei and increased GR expression in the hippocampus of a chronic unpredictable mild stress-induced depressive rat model [31]. Therefore, homeostasis of CRH receptors might be involved in improvement of the disequilibrium in the HPA system.

HPA hyperactivity was observed in 30–50% of all acutely depressed patients [32]. Mitochondrial dysfunctions affected important functions in MDD pathogenesis [23]. Small deletions of mitochondrial DNA were observed in muscles from patients with MDD [33]. Alterations in nuclear DNA-encoded mitochondrial mRNA and proteins in the cerebellum of MDD patients were also reported [34]. MDD patients with serious somatic complaints exhibited low ATP production rates in biopsied muscles [35]. These studies provide concrete evidence for the clinical relevance of an association between low ATP supply arising through mitochondrial dysfunction and MDD. XYS was reported by our group to ameliorate depressive-like behaviors in rats by regulating mammalian target of rapamycin (mTOR), suggesting that XYS may exert its anti-depressive effects through regulation of energy metabolism [36].

Inflammatory pathways were suggested to be involved in the pathophysiology of MDD through increased blood and cerebrospinal fluid concentrations of pro-inflammatory cytokines as well as acute phase proteins and their receptors [37]. Cytokines interact with mitochondria to increase the production of reactive oxygen species (ROS). Increased expressions of pro-inflammatory mediators, neurotoxic factors, and ROS contributed to the development of MDD [23]. XYS has been widely used for treating inflammatory diseases and depression comorbidities in hepatitis [38]. Recently, we found that XYS significantly reduced the serum levels of tumor necrosis factor-α and interleukin-6 in rats with depressive-like behaviors induced by chronic unpredictable mild stress (unpublished data). MDD was associated with neuronal atrophy and neuronal cell loss, especially in the hippocampus and cerebral cortex [39]. Decreased brain-derived neurotrophic factor (BDNF) was strongly associated with an increased risk for MDD [40]. A clinical meta-analysis showed that BDNF levels were associated with changes in depression [41]. BDNF was downregulated in the hippocampus of a CRS-induced rat depression model [42]. Reports from our group and others indicated that XYS increased BDNF expression in the hippocampus [36, 42, 43]. These results suggest that XYS improves MDD by upregulating BDNF in specific encephalic regions.

Epigenetic alterations were found in the frontal cortex of suicide victims with depression [44]. Antidepressants exerted some of their effects by causing epigenetic alterations [45]. Observed dysfunctions of biological clocks were related to MDD [46]. Patients with depression often showed altered circadian rhythms, sleep disturbances, and variations in diurnal moods [47]. The degree of circadian misalignment was correlated with the severity of depressive symptoms [47]. The actions of XYS on epigenetic modifications and circadian rhythms are significant, because this medicinal formula is efficacious in the treatment of sleeping and mood disorders.

### Integrated hypothesis for MDD as a unified mechanism of XYS

Hypotheses for MDD include 5-HT depletion, neurotrophin deficiency, neuroinflammation, mitochondrial dysfunction, HPA hyperactivity, epigenetic variation, and circadian dysrhythmia. However, the pathophysiology of MDD has rarely been studied and the published hypotheses are far from mutually exclusive.

The theory of inadequate monoamine neurotransmission, in which antidepressants increase monoamine availability and produce long-term adaptive changes in monoaminergic receptor sensitivity [48], is insufficient to explain MDD. Lowered plasma tryptophan reduced 5-HT synthesis and aggravated MDD symptoms [49]. *N*-acetylserotonin, an intermediate product of melatonin formation from 5-HT, is a specific agonist of BDNF receptors, and 5-HT is a substrate for melatonin biosynthesis. Melatonin deficiency contributed to primary and depression-associated insomnia as well as disturbances in circadian rhythms [50].

Neuroinflammation, which is characterized by increased production of interferon-γ, interleukin-6, and tumor necrosis factor-α, and induction of indoleamine 2,3-dioxygenase (IDO) in the blood and brain, plays a role in depression [37]. Activation of IDO reduces plasma tryptophan and brain 5-HT and increases the levels of tryptophan catabolites (TRYCATs), such as quinolinic and picolinic acids. Inflammation increases CRH and ACTH secretion. Cortisol levels are increased to activate liver tryptophan 2,3-dioxygenase, which further decreases plasma tryptophan and increases TRYCAT production. TRYCATs generate ROS, cause mitochondrial dysfunctions, and interfere with energy metabolism. They also potently activate NMDA receptors and induce pro-inflammatory responses and neuron apoptosis. These findings imply a shift from tryptophan and 5-HT depletion toward the detrimental effects of TRYCATs. IDO links the neuroinflammation and neurotoxicity of TRYCATs, which jointly promote the development of depressive symptoms [49].

Psychosocial stresses arising from life events can potentially induce continuous increases in stress hormones, which impair negative-feedback mechanisms and lead to continuous hyperactivity of the HPA axis. Pro-inflammatory cytokines are also potential activators of the HPA axis, thereby increasing the secretion of glucocorticoids, which are markers of glucocorticoid resistance. Glucocorticoids augment the alternative pathway for IDO-catalyzed tryptophan and decrease the amount of 5-HT available in synapses by increasing the expression of the serotonin transporter gene. Prolonged increases in glucocorticoids desensitize their receptors on immune cells, such as macrophages. Activation of macrophages in the periphery and brain occurred and pro-inflammatory cytokines were released in MDD patients [37]. Psychosocial stresses decrease the levels of BDNF and other neurotrophic/growth factors, while increasing the glucocorticoid concentration. Multiple interaction pathways exist between pro-inflammatory immune functions, brain and neuronal structures, brain serotonergic systems, and the HPA axis. The HPA axis is a key integrative component that links primary biological and psychosocial theories [36].

**Figure 2:**
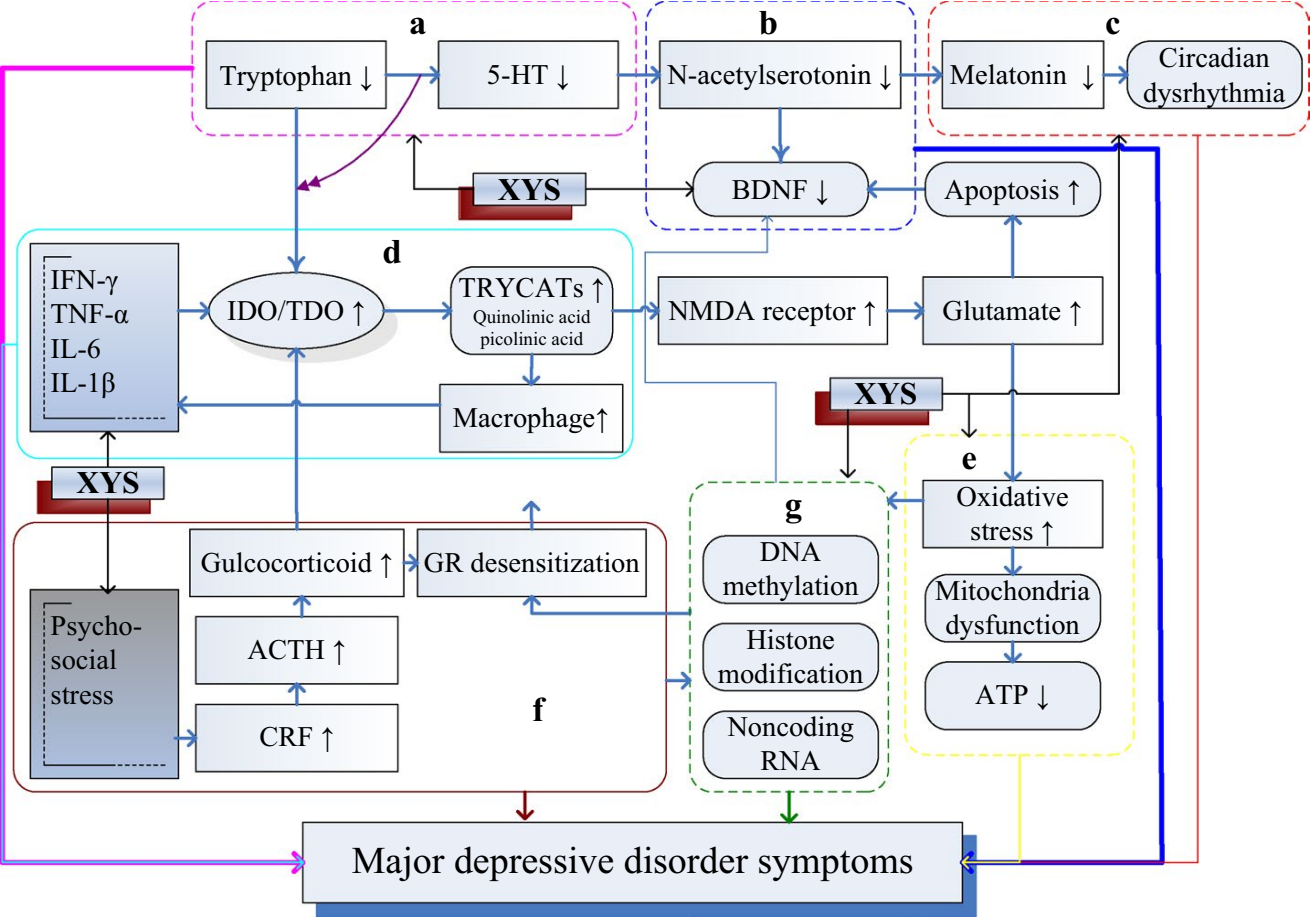
A simplified integrative model for the pathophysiology of MDD and the potential targets for XYS. **a**–**g** Refer to seven major hypotheses for MDD. **a** 5-HT depletion, **b** neurotrophin deficiency, **c** circadian dysrhythmia, **d** neuroinflammation, **e** mitochondria dysfunction, **f** HPA hyperacbivity, **g** epigenetic variation, *5-HT* 5-hydroxytryptamine, *BDNF* brain-derived neurotrophic factor, *IFN-γ* interferon γ, *TNF-α* tumor necrosis factor α, *IL-6* interleukin 6, *IDO* indoleamine 2,3-dioxygenase, *TDO* tryptophan 2,3-dioxygenase, *TRYCATs* tryptophan catabolites, *NMDA* *N*-methyl-d-aspartic acid, *GR* glucocorticoid receptor, *ACTH* adrenocorticotropic hormone, *CRH* corticotropin releasing hormone.

Dysfunction of the hippocampus, cerebellum, insula, frontal cortex, and temporal cortex could eventually contribute to the pathogenesis of MDD. The integrated model conjectures a general-purpose co-processor, whose effects depend on the specific brain centers to which individual modules are connected [51]. The disparate modules and different ideas on MDD emphasize the internal relationships among the different hypotheses (Figure 2).

## Future directions and implications

Although XYS is a widely used medicinal formula in China, the research results are not commensurable among the various modifications of XYS, i.e., different ingredients because of their origins, and forms of prescriptions, such as powders, decoctions, and pills. A new strategy for Chinese medicine quality control called formulomics was proposed to analyze XYS [52]. This strategy emphasizes strict extract quality control for XYS by liquid chromatography/mass spectrometry for chemical characterization and chemical fingerprinting [53], and recommends a fixed material origin and basis, and a standard extraction protocol. Targets for XYS in MDD must be screened with omics approaches and repeatedly confirmed using transgenic knockdown or knockin mice.

## Conclusions

The effectiveness of XYS for treatment of MDD is uncertain.

## Abbreviations

XYS: *Xiaoyaosan*
MDD: major depressive disorder
5-HT: 5-hydroxytryptamine
SSRIs: selective serotonin reuptake inhibitors
ROS: reactive oxygen species
HPA: hypothalamic–pituitary–adrenal
BDNF: brain-derived neurotrophic factor
IDO: indoleamine 2,3-dioxygenase
TRYCATs: tryptophan catabolites
CRH: corticotropin-releasing hormone
ACTH: adrenocorticotropic hormone
